# Whole genome re-sequencing reveals artificial and natural selection for milk traits in East Friesian sheep

**DOI:** 10.3389/fvets.2022.1034211

**Published:** 2022-10-18

**Authors:** Xiaolong Li, Lvfeng Yuan, Weimin Wang, Deyin Zhang, Yuan Zhao, Jiangbo Chen, Dan Xu, Liming Zhao, Fadi Li, Xiaoxue Zhang

**Affiliations:** ^1^College of Animal Science and Technology, Gansu Agricultural University, Lanzhou, China; ^2^Lanzhou Veterinary Research Institute, Chinese Academy of Agricultural Sciences, Lanzhou, China; ^3^The State Key Laboratory of Grassland Agro-ecosystems, College of Pastoral Agriculture Science and Technology, Lanzhou University, Lanzhou, China

**Keywords:** sheep, whole-genome resequencing, *F*_ST_, PI, sheep milk

## Abstract

The East Friesian sheep is one of the important high-yielding dairy sheep breeds, but still little is known about their genetic and genomic variation during domestication. Therefore, we analyzed the genomic data of 46 sheep with the aim of identifying candidate genes that are closely related to milk production traits. Our genomic data consisted of 20 East Friesian sheep and 26 Asian Mouflon wild sheep. Finally, a total of 32590241 SNPs were identified, of which 0.61% (198277) SNPs were located in exonic regions. After further screening, 122 shared genomic regions in the top 1% of *F*_ST_ and top 1% of Nucleotide diversity ratio were obtained. After genome annotation, these 122 candidate genomic regions were found to contain a total of 184 candidate genes. Finally, the results of KEGG enrichment analysis showed four significantly enriched pathways (*P* < 0.05): beta-Alanine metabolism (*SMOX, HIBCH*), Pathways in cancer (*GLI2, AR, TXNRD3, TRAF3, FGF16*), Non-homologous end-joining (*MRE11*), Epstein-Barr virus infection (*TRAF3, PSMD13, SIN3A*). Finally, we identified four important KEGG enrichment pathways and 10 candidate genes that are closely related to milk production in East Friesian sheep. These results provide valuable candidate genes for the study of milk production traits in East Friesian sheep and lay an important foundation for the study of milk production traits.

## Introduction

In recent years, the rapid development of next-generation sequencing (NGS) technology has provided important techniques and tools for the analysis of genetic traits. This development provides new opportunities to study economically important traits in animals (1–3). Recently, a number of population-related resequencing data studies have emerged that aim to identify important candidate genes for specific populations and specific traits. For example, For example, the identification of candidate genes and genomic intervals for heat-adapted traits in rainbow trout based on whole-genome resequencing data (4), the identification of candidate genes for litter size in goats (5), the characterization of lumbar vertebrae in sheep (6), and the study of candidate genes for adaptive capacity of yak populations to extreme environments (7). As well as a large number of studies that have used whole-genome retesting data to explore population genomic features and candidate genes in different species, such as pigs (8), dogs (9), chickens (10), and ducks (11).

The East Friesian sheep (EFS) is one of the most productive dairy sheep breeds, providing dairy products and by-products needed for daily human life. The East Friesian sheep lactation lasts about 230 days and produces 500–700 kg of milk in a single lactation (12). East Friesian sheep have been imported to many countries and are often used to improve native sheep in various countries due to their good milk and meat production performance (13). Although there have been reports on the use of East Friesian sheep as a research subject. For example, studies on wild and domesticated sheep (14), the effect of prolactin, β-lactoglobulin and kappa-casein genotypes on milk production in East Friesian sheep (15), studies on milk composition of different goat breeds (16), etc. However, little is known about the genetic and genomic variation associated with the East Friesian sheep population. To identify candidate genes for milk production traits in the East Friesian population more clearly, we used resequencing data from 20 East Friesian and 26 Asian Mouflon wild sheep (WS) to identify candidate genomic regions and candidate genes associated with milk production traits. The data included our own sequenced genomic data of 10 East Friesian sheep and genomic data of 10 East Friesian and 26 Asian Mouflon wild sheep from the NCBI SRA database (https://www.ncbi.nlm.nih.gov/sra). During the analysis, we used methods such as, phylogenetic tree analysis, population genetic structure analysis, principal component analysis (PCA), *F*_ST_, nucleotide diversity (Pi) and Kyoto Encyclopedia of Genes and Genomes (KEGG).

## Materials and methods

### Ethics statement

All experiments in this study were carried out in accordance with the approved guidelines from the Regulation of the Standing Committee of Gansu People's Congress. All experimental protocols, including the sample collection protocol, were approved by the Ethics Committee of Gansu Agriculture University (China) under permission no. DK-005.

### Samples and resequencing

We selected 10 East Friesian sheep (EFS) from Beijing Aoxin Animal Husbandry Co., LTD., China, for whole-genome resequencing (Supplementary Table S1) (17). The 5 ml blood sample was collected from the jugular vein of sheep, and the DNA was extracted by easy pure blood genomic DNA kit (TransGen biotech, Beijing, China), and then sequenced on Illumina nova seq 6000 (Illumina, San Diego, ca, United States). We also collected the resequencing data of 10 EFS and 26 Asian mouflon wild sheep (WS), which came from NCBI SRA database (https://www.ncbi.nlm.nih.gov/sra; Supplementary Table S3). Therefore, the resequencing data of 46 sheep were used for subsequent analysis.

### Alignment and SNP calling

The main data processing process is as follows: (1) Use Fastp (version: 0.12.4) software to filter the raw data with parameters: fastp --in1 A_1.fastq --in2 A_2.fastq --out1 A_clean_1.fastq --out2 A_clean_2.fastq -h A_fastp.html -j A_fastp.json (18). (2) Use BWA (Burrows-Wheeler Aligner, version: 0.7.15) software to match the filtered raw data to the reference genome (Oar_rambouillet_v1.0) with the parameters: bwa mem -R ‘@RG/tID:A/tSM:A' sheep1.0.fa A_clean_1.fastq A_clean_2.fastq > A.sam (19). (3) Use samtools (version: 1.10) software to first convert sam files into bam files, and then sort them by chromosome and locus with the following parameters: samtools view -b A.sam > A.bam; samtools sort -O BAM A.bam > A.sorted.bam (20). (4) Use samtools software to establish the index of the first Bam file, and then mark duplicates, parameters: samtools index A.sorted.bam; gatk MarkDuplicates -I A.sorted.bam -O A.dedup.bam -M A.mark_dup_metrics.txt; samtools index A.dedup.bam; samtools sheep1.0.fa; gatk CreateSequenceDictionary -R sheep1.0.fa. (5) Use GATK software to generate the gvcf file for each sample with the following parameters: gatk HaplotypeCaller -R sheep1.0.fa -I A.dedup.bam -O A.raw.gvcf -ERC GVCF -ploidy 2 -contamination 0 -G StandardAnnotation -G StandardHCAnnotation -G AS_StandardAnnotation -GQB 10 -GQB 20 -GQB 30 -GQB 40 -GQB 50 -GQB 60 -GQB 70 -GQB 80 -GQB 90 (21). (6) Use GATK software to merge all sample gvcf files into one gvcf file, parameter: gatk CombineGVCFs -R sheep1.0.fa -O combine_variants.raw.gvcf --variant A.raw.gvcf --variant B.raw.gvcf...... (7) Use GATK software to generate the original vcf file from the gvcf file with the following parameters: gatk GenotypeGVCFs -R sheep1.0.fa -O combine_variants.raw.vcf --variant combine_variants.raw.gvcf. (8) Use GATK software to generate SNP-only vcf files, parameter: gatk SelectVariants -R sheep1.0.fa -O combine_SNP.raw.vcf --variant combine_variants.raw.vcf --select-type-to-include SNP. (9) Use Vcftools software to filter vcf files, parameter: --remove-indels –min-allsles 2 –max-alleles 2 –minDP 4 –maxaDP 100 --minGQ 10 –minQ 30 –max-missing 0.6 –maf 0.05 (22). Finally, the ANNOVAR program (23) was used to annotate the filtered SNPs based on the sheep reference genome (Oar_rambouillet_v1.0), and finally the SNPs are divided into: exonic regions (variant overlaps a coding), intronic regions (variant overlaps an intron), upstream egions, downstream regions, and intergenic regions (variant is in the intergenic region).

### Phylogenetic and principal component analysis

A total of 32590241 high-quality SNPs were used for principal component analysis and phylogenetic tree construction. Use VCFtools (version: 0.1.16) to generate PLINK format files (22). Then, the PLINK (version: 1.9) program was used to generate principal component analysis (–out PCA_file) and phylogenetic tree (--distance --out tree_file) files (24). The phylogenetic tree uses the neighbor.exe subroutine in the phylip-3.698 program to generate a FigTree format file (https://evolution.genetics.washington.edu/phylip.html). Finally, FigTree was used to visualize the result file(http://tree.bio.ed.ac.uk/software/figtree/).

### Nucleotide diversity and selective sweep

We used the VCFtoos program to analyze nucleotide diversity (θ_π_) and fixation index (*F*_ST_; (25)). By comparing the differentiation index and nucleotide diversity of the EFS population and the WS population, the selected genomic intervals of the EFS population related to lactation traits were screened. We used VCFtools to calculate the *F*_ST_ value and θ_π_ value of the genome interval, the parameters:–fst-window-size 150000 –fst-window-step 75000, and calculate the θ_π_ ratio (θ_π*WS*_ / θπ*EFS*) (26). For the presentation of the theta π ratio, we use the y = log${}_{2}^{\text{x}}$ function to transform it and finally display it in the picture. In the two analysis methods of *F*_ST_ and θ_π_ ratio, the threshold line of each individual analysis is top 1%. Finally, visualization is performed using the R language packages ggplot2 (27) and ggExtra (28).

### Candidate interval and function annotation analysis

In order to find the genomic interval closely related to the lactation traits of the EFS population, we screened the interval shared by the top1% of the *F*_ST_ top1% and the θ_π_ ratio top1% of the genomic interval as candidate regions. ANNOVAR was used to annotate the SNPs in these regions to genes (23), and then Kyoto Encyclopedia of Genes and Genomes (KEGG) pathway analysis was performed on these candidate genes using KOBAS (29).

## Results

### Genomic variation

**Figure 1:**
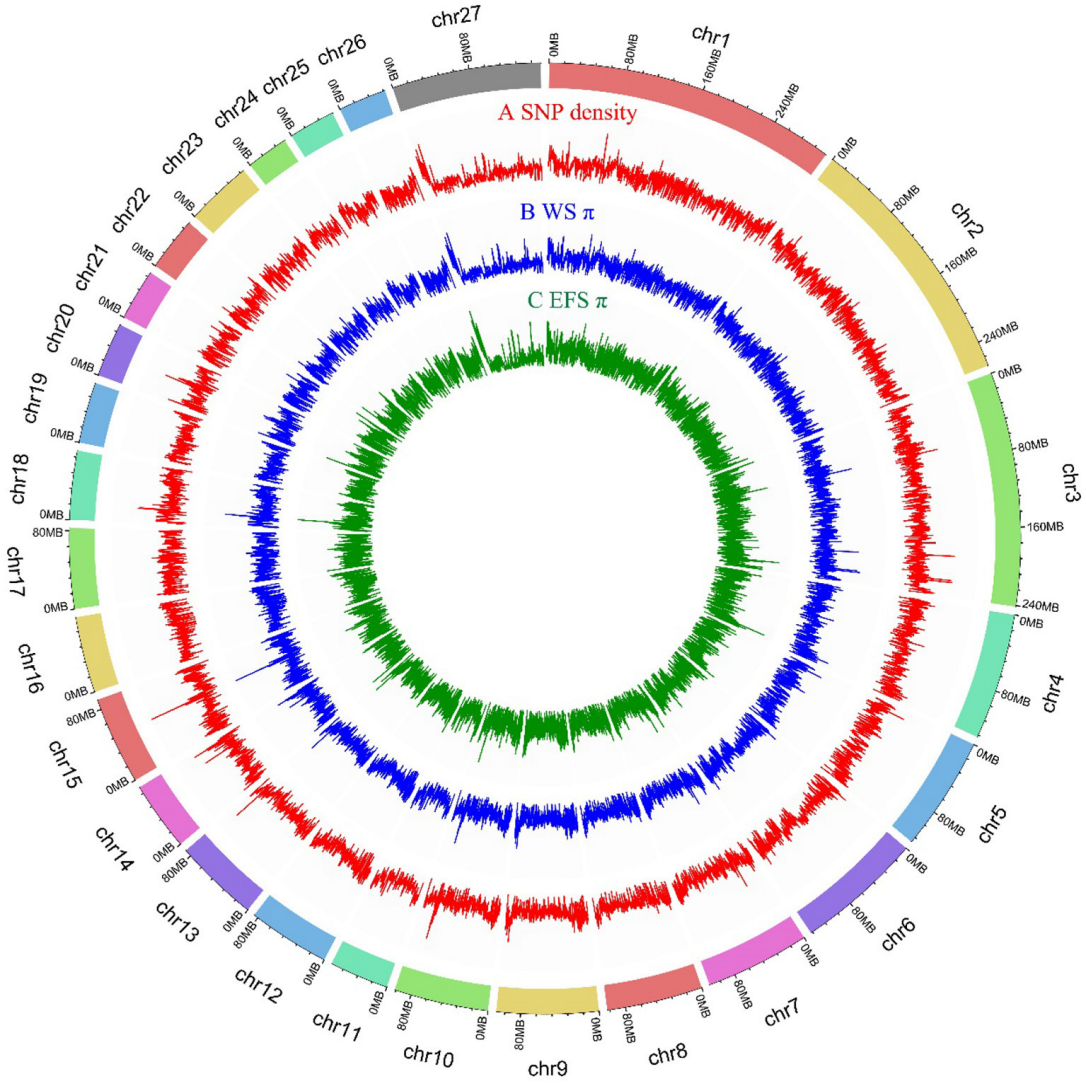
Genome-wide distribution of SNP density and PI in 150 kb size window. **(A)** Genome-wide distribution of SNP density within 150 kb window. **(B)** Genome-wide distribution of PI for WS population in 150 kb size window. **(C)** Genome-wide distribution of PI for EFS population in 150 kb size window.

We generated whole genome data for 10 EFS using Illumina NovaSeq 6000 instrument, and the statistical results revealed a total of 285.96 Gb of raw data, with an average sequencing depth of 10.6x (Supplementary Table S2). The data used in this study also included whole-genome sequencing data of 26 WS and 10 EFS from the NCBI database. Thus, the study used resequencing data from 46 sheep with a total data volume of 1.56 Tb. After rigorous matching and filtering, a total of 32590241 nucleotide polymorphisms were identified (Supplementary Table S4), of which 0.61% of SNP mutations were located in exonic regions (*N* = 198227), and the remaining SNP loci were mainly distributed in intergenic and intronic regions (94.41% of (Supplementary Table S5). The genome-wide 150 kb SNPs and PIs are depicted in Figure 1.

### Phylogenetic analysis and nucleotide diversity

**Figure 2:**
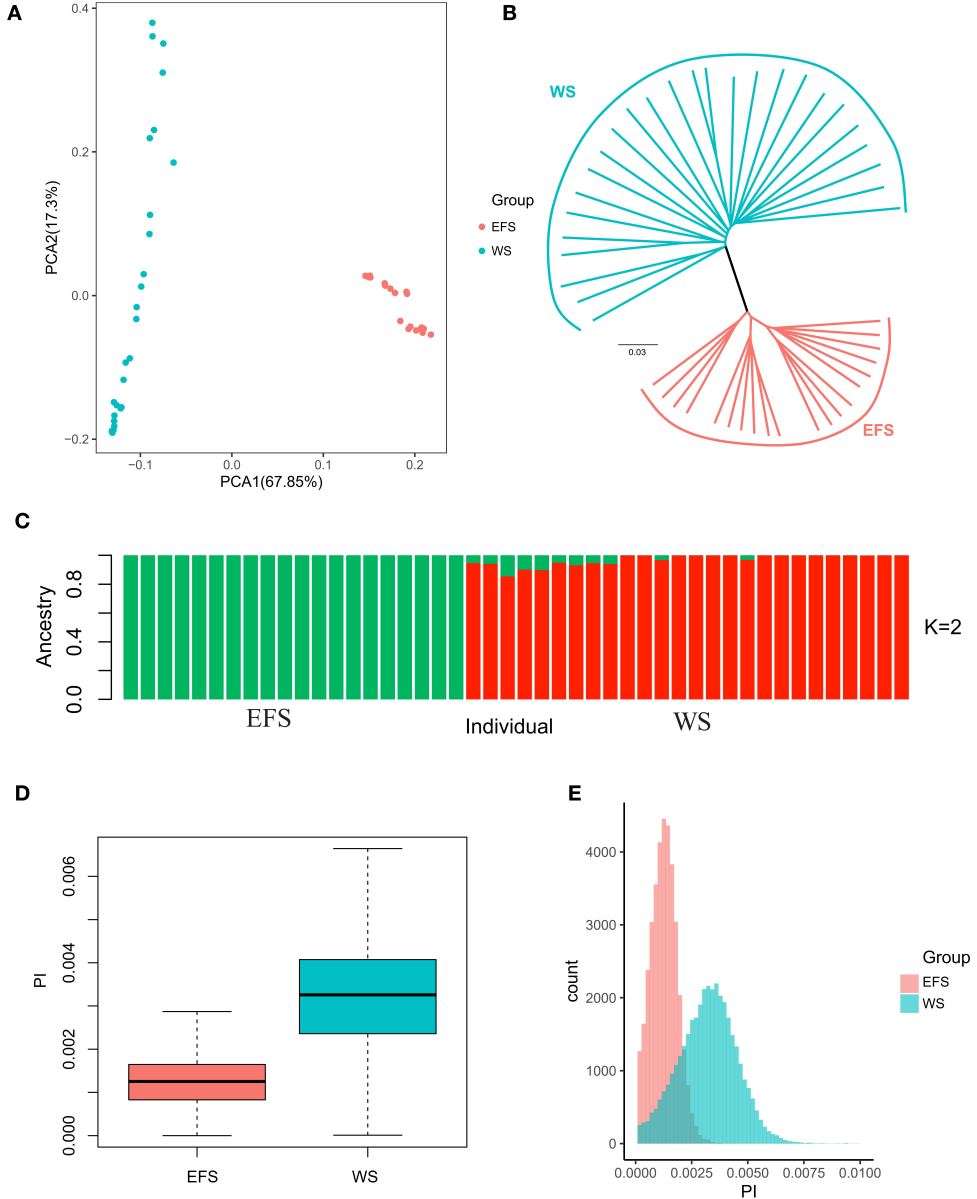
Phylogenetic and nucleotide diversity analysis. **(A)** PCA plots of EFS with the first two components of WS. **(B)** Phylogenetic tree of EFS and WS. **(C)** Box plot of nucleotide diversity of EFS and WS. **(D,E)** Nucleotide diversity density plot of EFS and WS.

Principal component analysis (PCA) was performed using the SNP dataset obtained above. The results showed that the first PCA axis clearly separated the EFS population from the WS population (Figure 2A). We then investigated the phylogenetic relationships between the EFS population and the WS population (Figure 2B). The results showed that the distance-based Neighbor-Joining tree was consistent with the results of the PCA analysis, both dividing the populations into the EFS and WS groups. Finally, similar results emerged in the analysis of the population genetic structure (Figure 2C). After calculating nucleotide diversity within a 150-kb window (step size 75 kb) for both groups (Supplementary Tables S6, S7), the EFS group (0.001250) showed lower nucleotide diversity than the WS group (0.003242) (Figures 2D,E).

### Genome-wide selection scans

**Figure 3:**
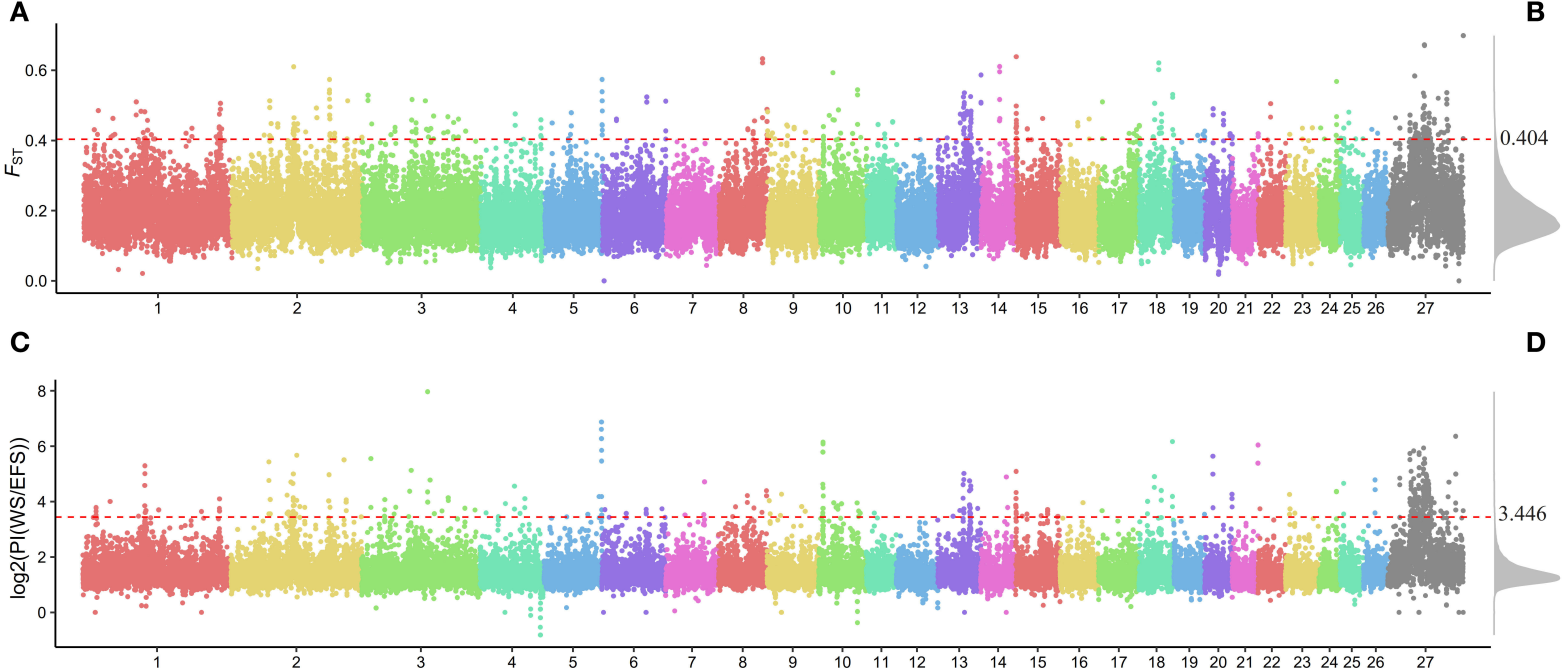
Genome-wide distribution of *F*_ST_ and *θ*_π_ ratio. **(A)** Genome-wide distribution of *F*_ST_. **(B)** Genome-wide density distribution of *F*_ST_. **(C)** Genome-wide distribution of PI [log2(PI(WS/EFS))]. **(D)** Genome-wide density distribution of PI. The horizontal red dashed line in the figure shows the window for the top 1% of *F*_ST_ and PI.

**Figure 4:**
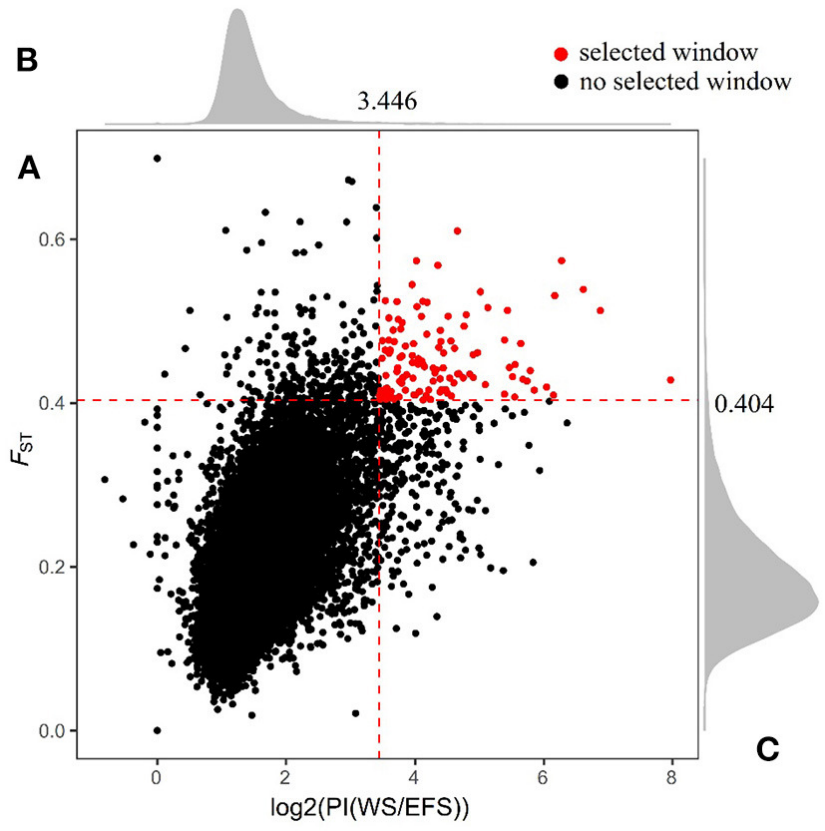
Shared genomic region of *F*_ST_ and *θ*_π_ ratio. **(A)** Window shared between *F*_ST_ and top 1% of PI. **(B)** Density distribution of PI in all Windows. **(C)** Density distribution of *F*_ST_ in all Windows. The red dashed line in the figure is the top 1% threshold line.

We calculated the fixed differentiation index (*F*_ST_; Supplementary Table S8) and the ratio of WS population to EFS nucleotide diversity θπ (θπWS / θπEFS) within a 150-kb window (step size 75 kb) (Supplementary Table S9), aiming to screen for positive selection windows associated with lactation traits. The top 1% window of each test performed was used as a candidate window (Figure 3). To further search for possible positive selection windows, we screened the shared windows between the top 1% of the *F*_ST_ and the top 1% of the θπ ratio as the final candidate windows. Finally, a total of 122 shared genomic regions were obtained (Figure 4, Supplementary Table S10). By annotation with ANNOVAR software, we obtained a total of 184 candidate genes from these 122 regions (Supplementary Table S11).

### Candidate genes and KEGG

**Figure 5:**
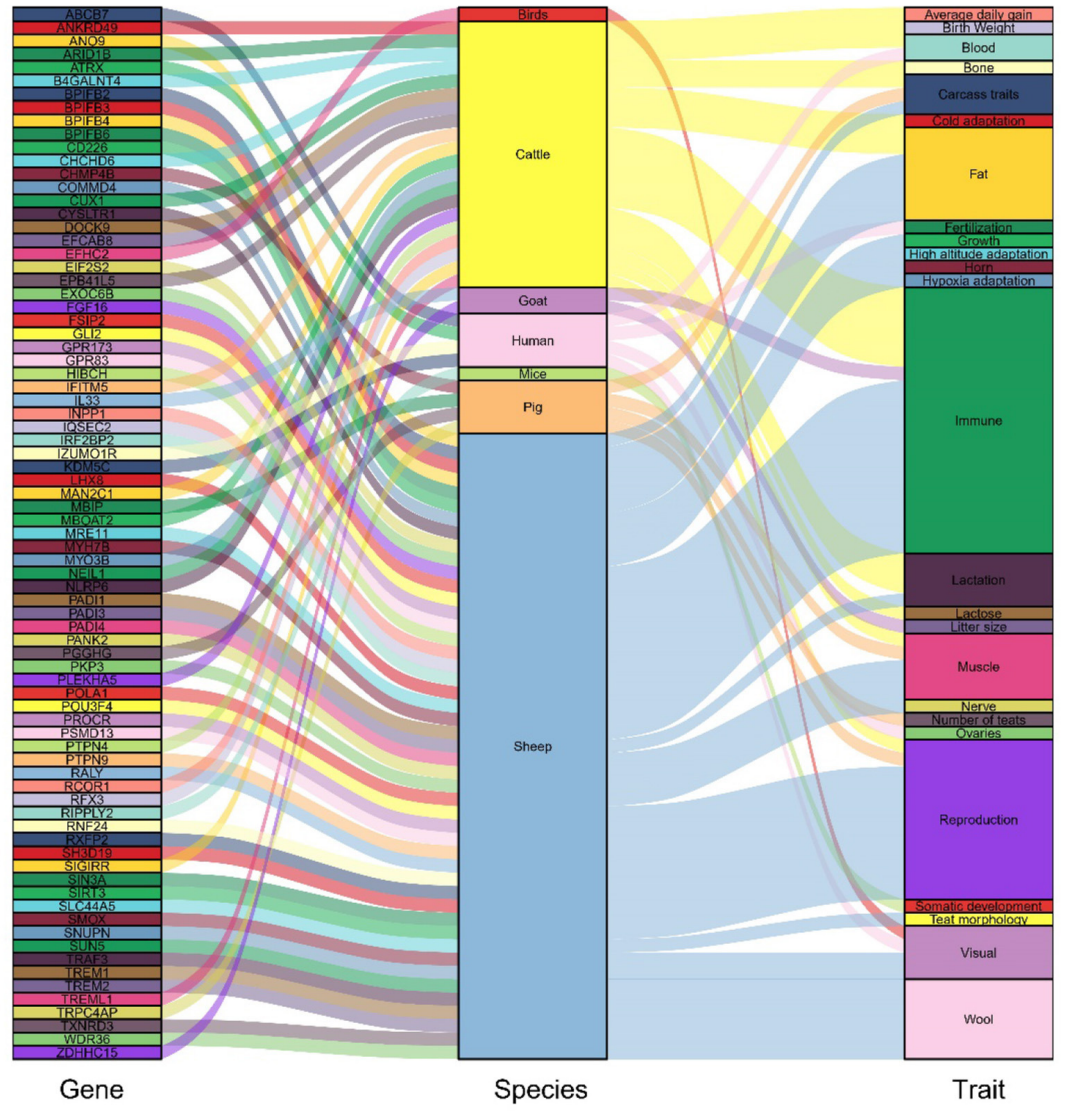
Candidate genes previously reported to be highly associated with domestication and its traits.

**Figure 6:**
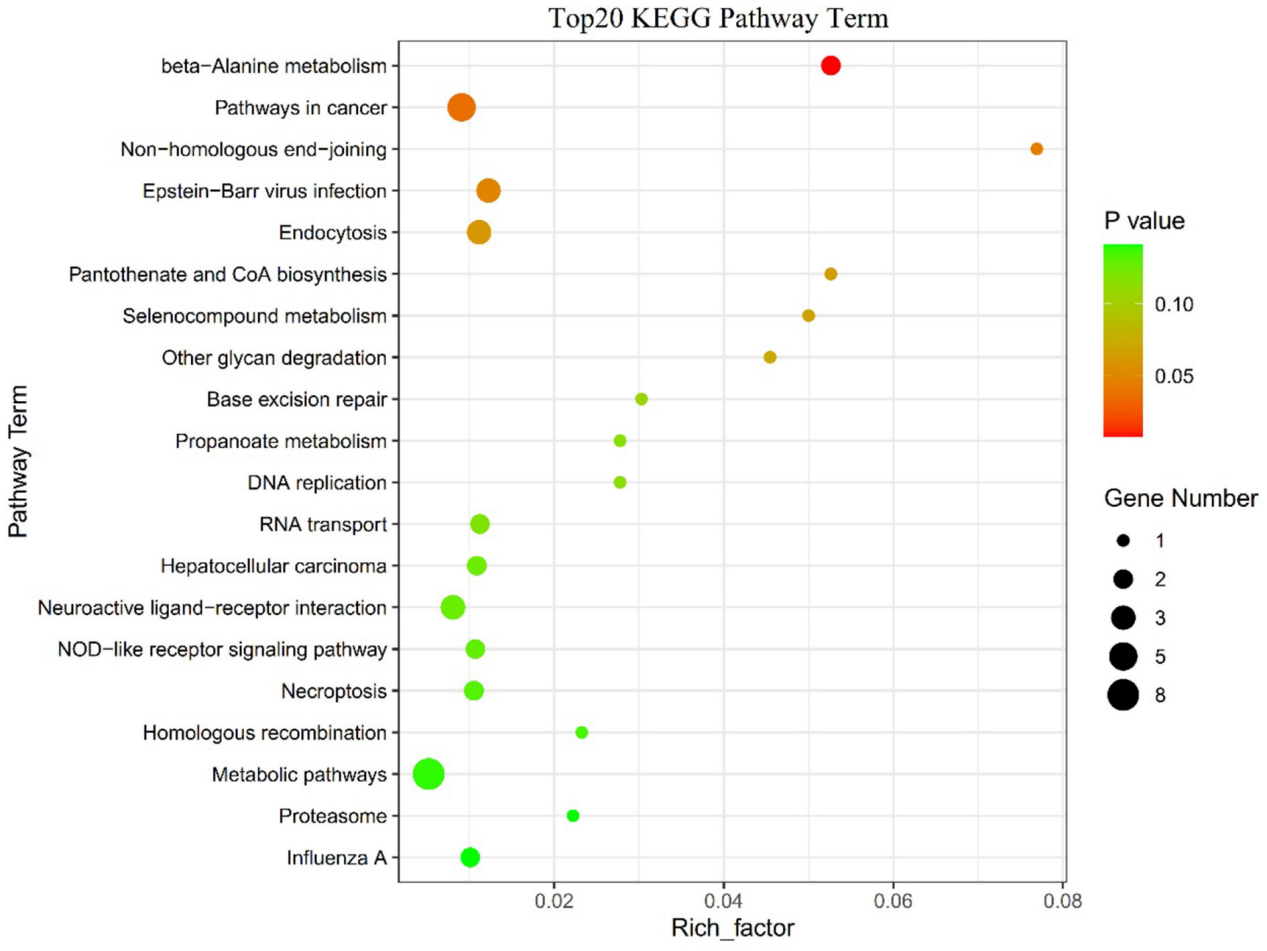
The top 20 KEGG enrichment pathways.

The SNPs in the 122 candidate windows obtained above were annotated to genes using ANNOVAR, and a total of 184 candidate genes were obtained (Supplementary Table S11). Included among these candidate genes are those screened for domestication-related candidates in previous reports (Figure 5, Supplementary Table S12). Then, these 184 genes were subjected to KEGG pathway enrichment analysis (Supplementary Table S13), and the top 20 enrichment pathways are shown in (Figure 6, Supplementary Table S14). Finally, four significant KEGG enrichment pathways were identified (*P* < 0.05, Table 1): beta-Alanine metabolism (*SMOX, HIBCH*), Pathways in cancer (*GLI2, AR, TXNRD3, TRAF3, FGF16*), Non-homologous end-joining (*MRE11*), Epstein-Barr virus infection (*TRAF3, PSMD13, SIN3A*).

**Table 1:** Four significantly enriched KEGG pathways.

**Related pathways**	***P*** **-value**	**Related genes**
beta-Alanine metabolism	0.00774	*SMOX, HIBCH*
Pathways in cancer	0.03702	*GLI2, AR, TXNRD3, TRAF3, FGF16*
Non-homologous end-joining	0.04524	*MRE11*
Epstein-Barr virus infection	0.04902	*TRAF3, PSMD13, SIN3A*

## Discussion

In this study, we performed whole-genome resequencing of 10 East Friesian sheep (EFS) and used whole-genome resequencing data of 10 East Friesian sheep (EFS) and 26 Asian mouflon wild sheep (WS) from the NCBI SRA database to develop subsequent analyses. We used a method similar to the previous study (30), compared the sequencing data to the sheep reference genome, and used various filtering methods (deep filtering, deletion rate and minimum allele frequency, etc.) to call the individual sheep SNP, to ensure that high-quality SNPs data are finally obtained. We identified a total of 32590241 high-quality SNPs from the use of genomic data, of which 0.61% (198277) SNPs are located in the exon region, which helps to reveal the genomic characteristics of the WS population and the EFS population.

The population genetic structure of EFS and WS was first analyzed, and the statistical results revealed that in the PCA analysis, PCA1 clearly divided the EFS and WS populations into 2 Clusters (Figure 2A). Immediately afterwards, in the phylogenetic tree (Figures 2B,C), the EFS and WS populations showed a similar classification pattern as in the principal component analysis, and this clustering pattern also appeared in previous studies (14). The nucleotide diversity of the two populations was found to be lower in the EFS population than in the WS population (Figures 2D,E), with an average nucleotide diversity (θπ) of 0.001250 in the EFS population and 0.003221 in the WS, which is consistent with previous studies that wild sheep have higher nucleotide diversity than other domesticated sheep breeds (14). This may be related to the complexity of the living environment of the Asiatic mouflon sheep, for example, the Asiatic mouflon sheep is one of the ancestors of the domesticated sheep, forced to forage, avoid natural enemies, adaptability of the environment and other pressures, the genome structure is diversified, and it produces a variety of proteins, only in this way can the Asiatic mouflon survive in the cruel environment of the wild, so the Asiatic mouflon shows a higher nucleotide diversity compared to the East Friesian sheep.

After screening the top 1% of genomic regions for *F*_ST_ (Figure 3A) and Pi ratio (Figure 3B), we then screened the genomic regions shared in these two methods and finally obtained 122 shared genomic regions (Figure 4). Among these 122 regions, we obtained a total of 184 candidate genes by annotation (Supplementary Table S11). Among these genes, we identified candidate genes that have been reported to be associated with economically important traits in animals (Figure 5, Supplementary Table S12). For example, in the reported studies in sheep, most of the genes were identified as important candidates associated with traits such as immunity, reproduction, vision, lipid synthesis, lactation, muscle differentiation, bone, blood, tail fat, wool and carcass. As well as in reported studies in cattle, pigs, goats, mice and birds, candidate genes associated with economically important traits such as daily weight gain, immunity, lactation, primordial weight, lactose synthesis, fat production, lambing numbers, muscle development, blood and cold environment adaptations also appeared in the results of this study. This demonstrates that the domestication and artificial selection of East Friesian dairy sheep is accompanied by simultaneous selection for multiple traits, and that the gradual increase in milk production is accompanied by simultaneous increases in traits such as immunity, neurodevelopment and metabolic levels.

Candidate genes previously reported to be associated with production traits appeared in the results of this study. For example, candidate genes related to lactation traits: *DOCK9, ARID1B, B4GALNT4, RCOR1, SH3D19*. The *DOCK9* gene was identified in a study on the impact of liver expression data on future lactation performance, showing that the *DOCK9* gene was differentially expressed and predicted to have an impact on the computational model of lactation performance (31). As well, *ARID1B* was identified as a significant differentially expressed gene in two groups of Holstein mammary glands with very high and very low percentages of milk protein and fat (32). The candidate genes *B4GALNT4* (33) involved in lactose synthesis, *RCOR1* (34) related to milk yield and *SH3D19* related to lactation process in sheep were also identified (35). In addition, our results contain a large number of candidate genes related to production traits, such as reproductive trait candidates: *FSIP2* (17), *CD226* (36), *SLC44A5* (37), *GPR83* (38), *SUN5* (39), *GPR173* (40), *LHX8* (41), *SIRT3* (42), *FGF16* (43), *POU3F4* (44), these genes affect reproductive traits by influencing days of estrus, luteal gland, participating in reproductive regulation, and hypothalamic-pituitary-gonadal axis regulation. For example, the candidate genes for wool traits: *RALY* (45), *GLI2* (46), *TXNRD3* (47), *EIF2S2* (45), *IRF2BP2* (48), etc.

Finally, the results of the KEGG enrichment analysis of genes in 122 regions showed four significantly enriched pathways (*p* < 0.05, Figure 6): beta-Alanine metabolism (*SMOX, HIBCH*), pathways in cancer (*GLI2, AR, TXNRD3, TRAF3, FGF16*), non-homologous end-joining (*MRE11*), Epstein-Barr virus infection (*TRAF3, PSMD13, SIN3A*). Therefore, we predict that these genes play an important role in lactation. For example, the *GLI2* gene was identified as a candidate gene for the Chronic subclinical mastitis trait in dairy cows, a disease that, when it occurs, can reduce milk production or lead to culling of cows (49). The *TRAF3* gene is essential for immune function of mammary epithelial cells in cows (50). The FGF16 gene was identified as differentially expressed in endometrial glandular, luminal, and stromal cells of cows with persistent inflammation and recovery from postpartum endometritis, demonstrating that the *FGF16* gene is a potential candidate gene to promote the transition from an inflammatory to a healthy state in postpartum dairy cows (51). As well, in a genome-wide association analysis of dairy cows, *SIN3A* was identified as a candidate gene for individual laboratory cheese yield and protein percentage traits in milk traits (52). Although some of these genes have been reported in dairy cattle studies, the biological functions of these candidate genes during milk production in sheep still need further validation.

## Conclusions

This is an analysis of genome-wide data from the East Friesian dairy sheep breed. We identified four important KEGG enrichment pathways and 10 candidate genes that are closely associated with milk yield in East Frisian sheep: beta-Alanine metabolism (*SMOX, HIBCH*), pathways in cancer (*GLI2, AR, TXNRD3, TRAF3, FGF16*), non-homologous end-joining (*MRE11*), Epstein-Barr virus infection (*TRAF3, PSMD13, SIN3A*). Some of these genes were identified as candidates for milk yield traits in dairy cattle studies, but the biological functions that these candidate genes play in milk production in sheep still need further validation. In conclusion, these results provide valuable candidate genes for the study of milk yield traits in East Friesian sheep and lay an important foundation for the study of milk yield traits.

## Data availability statement

The datasets presented in this study can be found in online repositories. The names of the repository/repositories and accession number(s) can be found below: https://www.ncbi.nlm.nih.gov/, SAMN22870744 - SAMN22870753 (17).

## Ethics statement

The animal study was reviewed and approved by Ethics Committee of Gansu Agriculture University (China) under permission no. DK-005. Written informed consent was obtained from the owners for the participation of their animals in this study.

## Author contributions

FDL, XXZ and WMW designed the experiments. XLL, XXZ, LFY and WMW analyzed the data. XLL wrote the manuscript. DYZ, YZ, JBC, DX and LMZ contributed to sample collection and prepared biological samples. XXZ, WMW and XLL revised the manuscript. All authors read and approved the final manuscript.

## Funding

This work was supported by the Earmarked Found for National Key R&D Program of China (2021YFD1300901), National Natural Science Foundation of China (31960653) and National Joint Research on improved breeds of Livestock and Poultry [grant no.19210365].

## Conflict of interest

The authors declare that the research was conducted in the absence of any commercial or financial relationships that could be construed as a potential conflict of interest.

## Publisher's note

All claims expressed in this article are solely those of the authors and do not necessarily represent those of their affiliated organizations, or those of the publisher, the editors and the reviewers. Any product that may be evaluated in this article, or claim that may be made by its manufacturer, is not guaranteed or endorsed by the publisher.
